# Real-world use of bempedoic acid for hypercholesterolaemia: a German multicentre, retrospective, cohort study

**DOI:** 10.1186/s12944-026-03062-6

**Published:** 2026-09-19

**Authors:** Linus Haberbosch, Andrea Baessler, Ulrike de Ruiter, David Sinning, Julius L. Katzmann, Stephanie Könemann, Ulrike Schatz, Knut Mai, Ursula Kassner, Thomas Bobbert

**Affiliations:** 1 Department of Endocrinology and Metabolism, Charité–Universitätsmedizin Berlin,Freie Universität Berlin, Humboldt-Universität zu Berlin, Berlin Institute of Health, European Reference Network on Rare Endocrine Diseases (ENDO-ERN), Charitéplatz 1, Berlin, 10117 Germany; 2 Department of Internal Medicine II, University Hospital Regensburg, Regensburg, Germany; 3 Dialysis and Lipid Centre Nordrhein, Essen, Germany; 4 ATOS MVZ KANT KARDIO, Berlin, Germany; 5 Department of Cardiology, University Hospital Leipzig, Leipzig, Germany; 6 Department of Internal Medicine B, University Medicine Greifswald, Greifswald, Germany; 7 DZHK (German Centre for Cardiovascular Research), Partner Site North, Fleischmann-Str. 42, Greifswald, 17475 Germany; 8 Department of Internal Medicine III, University Hospital Carl Gustav Carus, Dresden University of Technology, Dresden, Germany; 9 Department of Human Nutrition, German Institute of Human Nutrition, Potsdam- Rehbrücke, Nuthetal, Germany; 10 German Centre for Cardiovascular Research, Berlin, Germany; 11 German Centre for Diabetes Research, Neuherberg, Germany

**Keywords:** Dyslipidaemia, Bempedoic acid, Cardiovascular risk, Hypercholesterolaemia, Lipid lowering therapy, Low-density lipoprotein cholesterol

## Abstract

**Background:**

Dyslipidaemia remains a leading cause of death in Germany despite an improving therapeutic landscape, with many patients not achieving guideline-recommended low-density lipoprotein cholesterol (LDL-C) goals. Bempedoic acid, an adenosine triphosphate citrate lyase inhibitor, is a recent addition to the therapeutic landscape with proven efficacy in randomised controlled trials; however, its use varies between centres. We aimed to assess the short-term, real-world effectiveness of bempedoic acid in lowering LDL-C levels in patients in Germany.

**Methods:**

This retrospective, observational study was conducted using data from patients with primary hypercholesterolaemia or mixed dyslipidaemia, collected before and approximately 3 months after initiating bempedoic acid in 7 specialised lipid outpatient clinics in Germany.

**Results:**

Data were analysed from a total of 641 patients, with a mean age (standard deviation [SD]) of 63.2 (11.3) years. The addition of bempedoic acid significantly reduced LDL-C levels from a mean of 133.6 mg/dL at baseline to 101.8 mg/dL after 3 months (p < 0.001). The mean within-patient LDL-C reduction was 31.8 mg/dL and the mean patient-level percentage change was -17.5%. Mean patient-level changes were -4.9% for HDL-C and -12.0% for total cholesterol; triglycerides did not change significantly. At 3 months, patients not receiving any lipid-lowering therapy (LLT) other than bempedoic acid had a mean patient-level LDL-C reduction of 27.1%; corresponding mean reductions were 23.7% with ezetimibe, 20.3% with PCSK9 inhibitor therapy, 9.0% with statin therapy and 6.1% with statin plus ezetimibe. Suspected adverse events were documented in 218/641 patients (34.0%), and 171/641 (26.7%) had discontinued bempedoic acid by follow-up. Myalgia was the most commonly reported adverse event in patients discontinuing treatment (75 cases).

**Conclusions:**

Findings from this real-world, multicentre study conducted in Germany show that bempedoic acid may present an effective treatment option for patients with hypercholesterolaemia who are already receiving LLT, in addition to being an effective option for patients not receiving/tolerating other LLTs. The magnitude of LDL-C reduction differed across background treatment strategies, while suspected adverse events and treatment discontinuation were common in this specialist-care cohort.

## Background

Cardiovascular (CV) disease is the leading cause of death in Germany, accounting for around 40% of all deaths [1]. It is widely recognised that high levels of low-density lipoprotein cholesterol (LDL-C) are associated with an increased risk of atherosclerotic cardiovascular disease (ASCVD) [2]. The effect of LDL-C on CV risk seems to depend on both the magnitude and the duration of exposure to LDL-C [3], with a reduction of 1 mmol/L (39 mg/dL) in LDL-C being associated with a 22% risk reduction in major vascular events per year [4]. Therefore, reducing LDL-C levels plays an important role in lowering the risk of CV disease.

The European Society of Cardiology (ESC)/European Atherosclerosis Society (EAS) 2019 joint guidelines for the management of dyslipidaemia recommend an LDL-C reduction of ≥ 50% from baseline, as well as an absolute LDL-C level of ˂ 1.8 mmol/L (< 70 mg/dL) for patients at high CV risk and ˂ 1.4 mmol/L (< 55 mg/dL) for those at very high CV risk, to reduce the risk of ASCVD [2]. The International Lipid Expert Panel (ILEP) has also introduced a definition for an ‘extremely high risk’ group of patients following acute coronary syndrome (ACS), which recommends a lower LDL-C target of ˂1.0 mmol/L (< 40 mg/dL) [5], which is similarly recommended in the recent ESC/EAS dyslipidaemia guidelines update for patients with progressive or polyvascular atherosclerotic disease [6]. However, many patients do not achieve these guideline-recommended LDL-C goals [7].

The ESC/EAS 2019 guidelines for the pharmacological lowering of LDL-C recommend that a high-intensity statin at the highest tolerated dose to reduce incident and recurrent ASCVD events, in addition to lifestyle modification. If goals are not achieved, combination therapy with ezetimibe is recommended, and for some patients, a proprotein convertase subtilisin/kexin type 9 inhibitor (PCSK9i) may be considered. Since 2021, upfront (immediate) lipid-lowering combination therapy has been used to achieve LDL-C goals in very high-risk and extremely high-risk patients [2]. The 2023 ESC guidelines allow for upfront lipid-lowering combination therapy in patients with ACS [8, 9]. Furthermore, the 2024 ILEP position paper recommends upfront lipid-lowering combination therapy in very high-risk and extremely high-risk patients [8], similarly to the 2025 ESC/EAS guidelines update, recommending upfront combination therapy in patients with ACS unlikely to achieve the treatment target with statin monotherapy [6].

Bempedoic acid is a first-in-class adenosine triphosphate citrate lyase inhibitor [10], and has demonstrated efficacy in reducing LDL-C levels and the risk of CV events in the CLEAR clinical trial programme [11–14]. Bempedoic acid and a bempedoic acid + ezetimibe fixed-dose combination have been approved by the European Medicines Agency for the treatment of primary hypercholesterolaemia and mixed dyslipidaemia, and for the reduction of CV risk by lowering LDL-C levels [15, 16]. The 2025 update of the ESC/EAS 2019 guidelines recommend the use of bempedoic acid for patients whose condition does not respond to statins to reach their LDL-C goals [6]. For patients who do not reach their LDL-C goals with statins and ezetimibe, the addition of bempedoic acid should be considered [6]. However, its use varies widely between centres, and real-world data on its effectiveness and safety are limited.

The evolving role of bempedoic acid in contemporary dyslipidaemia management, including its use in statin-intolerant patients and in combination lipid-lowering strategies, has also been reviewed and addressed in recent expert guidance [17, 18].

In this multicentre, retrospective analysis conducted in Germany, we aimed to assess the short-term, real-world effectiveness of bempedoic acid in lowering patients’ LDL-C levels. We additionally characterized background LLT patterns, treatment persistence and suspected adverse events in routine specialist care.

## Methods

### Study design and patient population

This retrospective, observational study was conducted using data from patients with primary hypercholesterolaemia or mixed dyslipidaemia, collected from electronic or physical records between 1 November 2020 and 30 April 2024 at 7 specialised lipid outpatient clinics in Germany. Patients were eligible for inclusion if they were 18–80 years of age, had a dyslipidaemia diagnosis and had initiated bempedoic acid during the study period and had at least LDL-C measurements available at the specified timepoints. Patients received treatment for lipid lowering for primary or secondary cardiovascular prevention. There were no exclusion criteria. Patients who discontinued bempedoic acid before the follow-up assessment remained eligible for analysis. A dedicated variable reliably distinguishing heterozygous familial hypercholesterolaemia from non-familial hypercholesterolaemia was not available in the analytic dataset; familial status was therefore not inferred retrospectively. The requirement for individual informed consent was waived by the ethics committee because the study used retrospectively collected routine clinical data.

Lipid profile data (LDL-C, triglycerides [TG], high-density lipoprotein cholesterol [HDL-C] and total cholesterol [TC]) were collected before bempedoic acid initiation and from a routine clinical follow-up visit at 3-month, with a tolerance window of +/- 30 days. Exact treatment-initiation and follow-up dates were not available in the analytic dataset, so the elapsed follow-up interval in days could not be reconstructed.

Cardiovascular risk was characterized in an exploratory post hoc analysis using unequivocal criteria available in the dataset. Documented ASCVD was considered a very-high-risk criterion; among patients without documented ASCVD, eGFR 30–59 mL/min/1.73 m2 or baseline LDL-C > = 190 mg/dL was used to identify high-risk patients. Patients not reliably classifiable from the available variables were left unclassified. Absolute LDL-C goal attainment was defined as LDL-C < 55 mg/dL for very-high-risk and < 70 mg/dL for high-risk patients; the > = 50% reduction-from-untreated-baseline criterion could not be assessed uniformly because untreated LDL-C was not consistently available.

### Objectives

The primary objective was the change in LDL-C levels from pre-treatment to follow-up in patients receiving bempedoic acid. Secondary objectives included reported adverse drug reactions, treatment discontinuations and reasons for discontinuation.

### Statistical analysis

All data were analysed descriptively, with results presented as mean and standard deviation (SD) where appropriate; triglycerides were summarized using median and interquartile range and analysed with non-parametric tests because of their skewed distribution. Adverse events analysed by co-medication combination at baseline excluded combinations received by fewer than 50 patients. Lipid changes were calculated within each patient before summary.

The analysis used retrospectively available routine clinical records from the participating centres. A separate screening log was not available; therefore, the number of potentially eligible patients who were not included in the analytic dataset could not be reconstructed.

To assess changes in lipid profile, we employed a multivariate general linear model of within-patient changes with LDL-C, HDL-C, TC and TG as dependent variables, controlling for age and sex; complete-case multivariate analyses included 557 patients. The adjusted overall multivariate change and associations of change with age and sex were assessed using Pillai’s trace, followed by univariate models for each lipid parameter. Between-subject associations of age and sex with average lipid levels across baseline and follow-up were additionally assessed. Complete-case analysis required baseline and follow-up measurements for all four lipid parameters together with available age and sex. Percentage change for each lipid parameter was calculated separately within patient as 100 x (follow-up value - baseline value)/baseline value and then summarised across patients with paired measurements; missing lipid values were not imputed. Additionally, an exploratory one-way analysis of variance (ANOVA) was conducted to examine differences in patient-level percentage LDL-C change across the five most common co-medication combinations (no concomitant LLT, statin monotherapy, ezetimibe monotherapy, statin + ezetimibe, and PCSK9i monotherapy). Follow-up LDL-C was additionally compared across these groups using ANCOVA adjusted for baseline LDL-C, age and sex, with Bonferroni-adjusted pairwise comparisons. Dunnett-adjusted contrasts compared each group’s mean patient-level LDL-C reduction with the no-concomitant-LLT reference. A post hoc mechanism-informed analysis compared patients without a background cholesterol-synthesis inhibitor (no concomitant LLT, ezetimibe or PCSK9 inhibitor) with patients receiving statin-containing background therapy (statin or statin plus ezetimibe); Welch’s t test was used for the primary percentage-reduction comparison, with Mann-Whitney testing and endpoint ANCOVA adjusted for baseline LDL-C, age and sex as sensitivity analyses. Suspected adverse events documented in routine clinical follow-up were retrospectively extracted from the source medical records and entered into the study dataset using predefined variables. Standardised study-data extraction was used to improve consistency of retrospective coding; however, adverse events were not ascertained using a uniform prospective surveillance protocol. These data were analysed descriptively. Statistical analyses were performed using IBM SPSS Statistics Version 29.0 (IBM) and RStudio Version 2024 (Posit Software). Figures were created in RStudio and GraphPad Prism Version 10. Significance levels were set at *p* < 0.05.

## Results

In total, 641 patients were analysed. The mean (SD) age was 63.2 (11.3) years, 58.7% of patients were female (Table 1), 18.4% had type 2 diabetes and 43.0% were receiving statins at baseline (Table 2). The most common lipid-lowering therapies (LLTs) at baseline were ezetimibe (47.0%), statins (43.0%) and PCSK9is (16.3%) (Table 2). Baseline LLT was summarized by individual drug class in this Table. These drug-class categories are not mutually exclusive, as a patient receiving combination therapy is counted in each applicable drug-class category. For the exploratory LDL-C subgroup analysis, patients were separately assigned to mutually exclusive categories representing the five most common concomitant treatment patterns. Documented hypercholesterolaemia was present in 584/641 patients (91.1%) and mixed dyslipidaemia/combined hyperlipoproteinaemia in 199/641 (31.0%); these diagnostic variables were not mutually exclusive. Using the available unequivocal risk features, 484/641 patients (75.5%) met a very-high-risk criterion, 50/641 (7.8%) met a high-risk criterion, and 107/641 (16.7%) could not be reliably classified. Previous discontinuation because of documented intolerance was recorded for statins in 190/640 patients (29.7%), ezetimibe in 119/641 (18.6%), PCSK9 inhibitors in 6/639 (0.9%), fibrates in 11/639 (1.7%) and bile acid sequestrants in 3/639 (0.5%).

**Table 1:** Baseline demographics and clinical characteristics.

Characteristic	Mean (SD)*	*n* (*N* = 641)
Age, years	63.2 (11.3)	641
Sex		
Female, %	58.7	376
Male, %	41.3	265
BMI, kg/m^2^	27.5 (4.9)	363
HbA1c, %	6.0 (0.7)	280
Creatinine, mg/dL	0.9 (0.4)	449
eGFR, mL/min/1.73m^2^	78.0 (16.5)	446
AST, U/L	29.2 (11.1)	346
ALT, U/L	30.2 (18.3)	409
GGT, U/L	36.5 (36.0)	400
CK, U/L	140.4 (171.4)	368
Thyroid-stimulating hormone, mU/L	2.5 (7.5)	181
Haemoglobin, g/dL	14.0 (2.4)	230
Platelets, 10^3^/µL	249.0 (55.9)	210
Glucose, mg/dL	102.6 (33.8)	335
Uric acid, mg/dL	5.5 (3.9)	371

*Mean (*SD*) is presented unless otherwise specified *ALT* Alanine transaminase, *AST* Aspartate aminotransferase, *BMI* Body mass index, *CK* Creatinine kinase, *eGFR* Estimated glomerular filtration rate, *GGT*, Gamma-glutamyl transferase *HbA1c* Glycated haemoglobin, *SD* Standard deviation

**Table 2:** Prior cardiometabolic disease and lipid-lowering therapy at baseline

*n*/*N* (%)	Yes	No	Unknown
Medical conditions			
Cardiovascular disease	265/638 (41.5)	353/638 (55.3)	20/638 (3.1)
Myocardial infarction	121/641 (18.9)	492/641 (76.8)	28/641 (4.4)
Stroke	42/639 (6.6)	573/639 (89.7)	24/639 (3.8)
Other atherosclerosis	281/639 (44.0)	332/639 (52.0)	26/639 (4.1)
Hypertension	408/640 (63.8)	224/640 (35.0)	8/640 (1.3)
Type 2 diabetes	117/637 (18.4)	509/637 (80.0)	11/637 (1.7)
Obesity	120/641 (18.7)	502/641 (78.3)	19/641 (3.0)
Heart failure	66/639 (10.3)	532/639 (83.3)	41/639 (6.4)
Chronic kidney disease	122/639 (19.1)	503/639 (78.7)	14/639 (2.2)
Hyperuricaemia	46/637 (7.2)	554/637 (87.0)	37/637 (5.8)
	Yes	No	Previously discontinued because of intolerance
Lipid-lowering therapy*			
Statins	275/640 (43.0)	175/640 (27.3)	190/640 (29.7)
Ezetimibe	303/641 (47.3)	219/641 (34.2)	119/641 (18.6)
PCSK9i	104/639 (16.3)	529/639 (82.8)	6/639 (0.9)
Inclisiran	8/638 (1.2)	630/638 (98.7)	0/638 (0.0)
Fibrate	19/639 (3.0)	609/639 (95.3)	11/639 (1.7)
Omega-3 fatty acid supplements	15/638 (2.4)	623/638 (97.6)	0/638 (0.0)
Bile acid sequestrants	21/639 (3.3)	615/639 (96.2)	3/639 (0.5)

Percentages rounded to one decimal place. Differing values for N are due to missing data. *Drug-class rows are not mutually exclusive across therapies; current use includes use as part of combination therapy. The ‘previously discontinued because of intolerance’ column reflects the corresponding labelled category in the source data. PCSK9i, proprotein convertase subtilisin/kexin type 9 inhibitor

In the complete-case multivariate analysis (*n* = 557), there was a significant overall within-patient change in the lipid profile from baseline to follow-up (Pillai’s trace = 0.333, F[4, 551] = 68.80, *p* < 0.001), after accounting for age and sex. Follow-up univariate analyses of the change scores showed significant changes in LDL-C (F[1, 554] = 230.86, *p* < 0.001), HDL-C (F[1, 554] = 40.95, *p* < 0.001), and TC (F[1, 554] = 205.90, *p* < 0.001), whereas the change in TG was not statistically significant (F[1, 554] = 2.81, *p* = 0.094). (Table 3).

**Table 3:** Lipid profiles at baseline and 3-month follow-up

Paired values [*n*]	Baseline	Follow-up	Change
LDL-C, mg/dL	133.6 (60.3) [*n* = 641]	101.8 (46.0) [*n* = 641]	-31.8 mg/dL; -17.5%
Triglycerides, mg/dL	135.0 (96.5–188.0) [*n* = 579]	128.0 (85.0-187.0) [*n* = 579]	-3.5%*
HDL-C, mg/dL	59.4 (17.9) [*n* = 587]	56.0 (17.5) [*n* = 587]	-3.3 mg/dL; -4.9%
Total cholesterol, mg/dL	213.5 (67.8) [*n* = 589]	181.1 (53.8) [*n* = 589]	-32.4 mg/dL; -12.0%

*HDL-C* High-density lipoprotein cholesterol, *LDL-C* Low-density lipoprotein cholesterol,* SD* Standard deviation

*Triglycerides are median (IQR) and were compared using a paired Wilcoxon signed-rank test (p = 0.132). All other values are mean (SD) and paired comparisons were p < 0.001. Percentage changes were calculated within patient before summarization

Age was significantly associated with the multivariate pattern of within-patient lipid changes (Pillai’s trace = 0.022, F[4, 551] = 3.05, *p* = 0.017). In the univariate analyses, increasing age was associated with smaller reductions in LDL-C (*p* = 0.021) and TC (*p* = 0.046), and greater reductions in TG (*p* = 0.036), while showing no significant association with HDL-C (*p* = 0.064). Sex was not significantly associated with the overall multivariate pattern of lipid changes (Pillai’s trace = 0.013, F[4, 551] = 1.86, *p* = 0.116), although an association with TG change was observed in the univariate analysis (F[1, 554] = 4.16, *p* = 0.042), with male patients showing a more positive TG change than female patients.

Between-subject analyses of mean lipid levels across baseline and follow-up showed significant associations with sex for all lipid measures (all *p* < 0.001). Specifically, female patients had higher mean LDL-C, HDL-C and TC levels, whereas male patients had higher mean TG levels. Increasing age was significantly associated with higher mean HDL-C: F(1, 554) = 13.43, *p* < 0.001, and lower mean TG: F(1, 554) = 8.57, *p* = 0.004, but not with LDL-C or TC.

Numerical changes in lipid profiles at 3-month follow-up are shown in (Table 3).

**Fig. 1:**
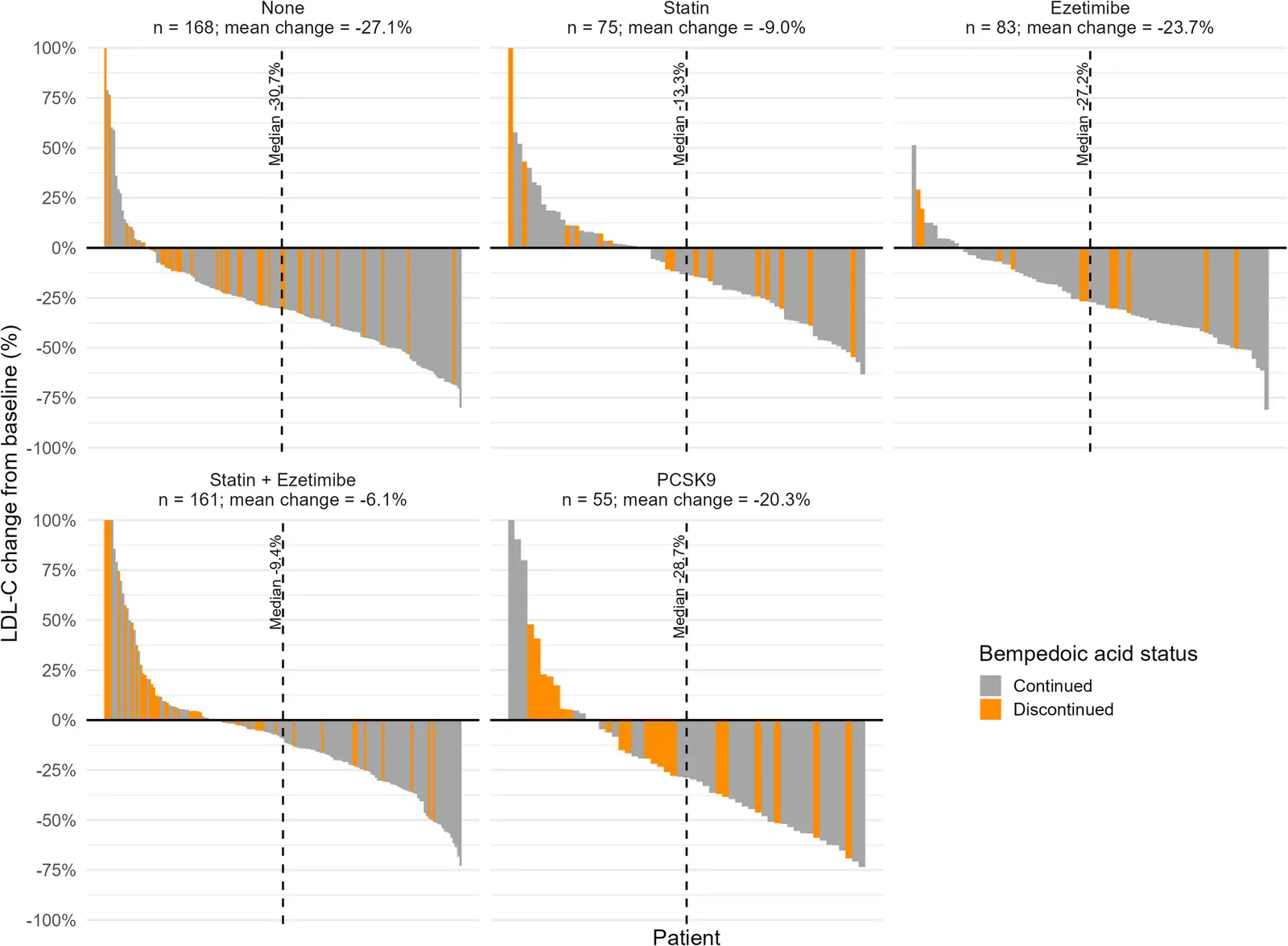
Individual percentage change in LDL-C from baseline to follow-up according to concomitant lipid-lowering therapy. Each bar represents one patient; negative values indicate LDL-C reduction. The dashed vertical line in each panel marks the median-ranked response and its label reports the subgroup median percentage change. For visual clarity, increases >100% are displayed at the +100% plotting boundary; observed values >100% were: no concomitant LLT 249%; statin 181%; statin plus ezetimibe 192%, 159%, 131% and 124%; PCSK9 inhibitor 176%. All original values were retained in statistical analyses. The figure displays the five most common concomitant-LLT patterns (n = 542); 99 patients receiving other combinations are not shown

**Fig. 2:**
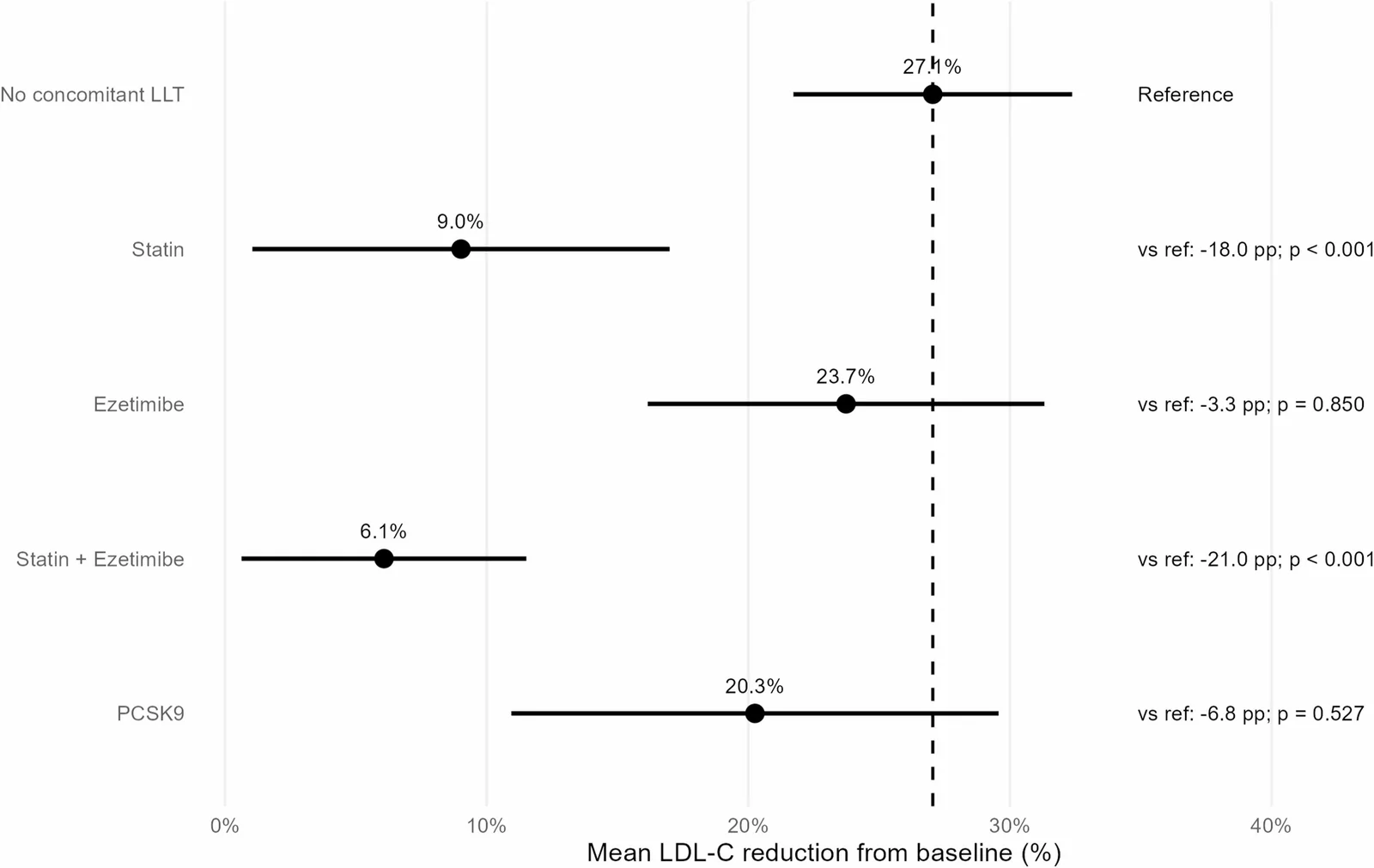
Relative LDL-C reduction by concomitant lipid-lowering treatment group. Points show mean patient-level LDL-C reduction with 95% confidence intervals, displayed as a positive percentage. No concomitant LLT is the reference; annotations report the difference in mean reduction versus reference in percentage points and Dunnett-adjusted p values

In an exploratory ANOVA, the patient-level percentage change in LDL-C was found to differ significantly across medication groups (F[4, 537] = 9.16, *p* < 0.001). The largest LDL-C reductions were observed in patients receiving no concomitant LLTs, with a mean patient-level LDL-C reduction of 27.1% (*n* = 168). Mean reductions were 9.0% with statin therapy (*n* = 75), 23.7% with ezetimibe (*n* = 83), 6.1% with statin plus ezetimibe (*n* = 161), and 20.3% with PCSK9 inhibitor therapy (*n* = 55) (Fig. 1). Using no concomitant LLT as the reference, mean relative LDL-C reduction was significantly smaller with statin therapy (difference − 18.0% points, Dunnett-adjusted *p* < 0.001) and statin plus ezetimibe (-21.0% points, *p* < 0.001), but did not differ significantly with ezetimibe (-3.3% points, *p* = 0.850) or PCSK9 inhibitor therapy (-6.8% points, *p* = 0.527) (Fig. 2). Baseline LDL-C differed markedly across groups; after adjustment for baseline LDL-C, age and sex, the overall co-medication effect on follow-up LDL-C was of borderline statistical significance (F[4,534] = 2.34, *p* = 0.054), with no Bonferroni-adjusted pairwise comparison reaching *p* < 0.05.

The no-concomitant-LLT group comprised 168 patients; mean age was 63.8 years, 63.7% were female, mean baseline LDL-C was 180.8 mg/dL, 66.1% had documented ASCVD, 20.2% had type 2 diabetes, 23.8% had renal insufficiency, 35.1% had mixed dyslipidaemia, and 52.4% had previously discontinued statin therapy because of documented intolerance.

**Fig. 3:**
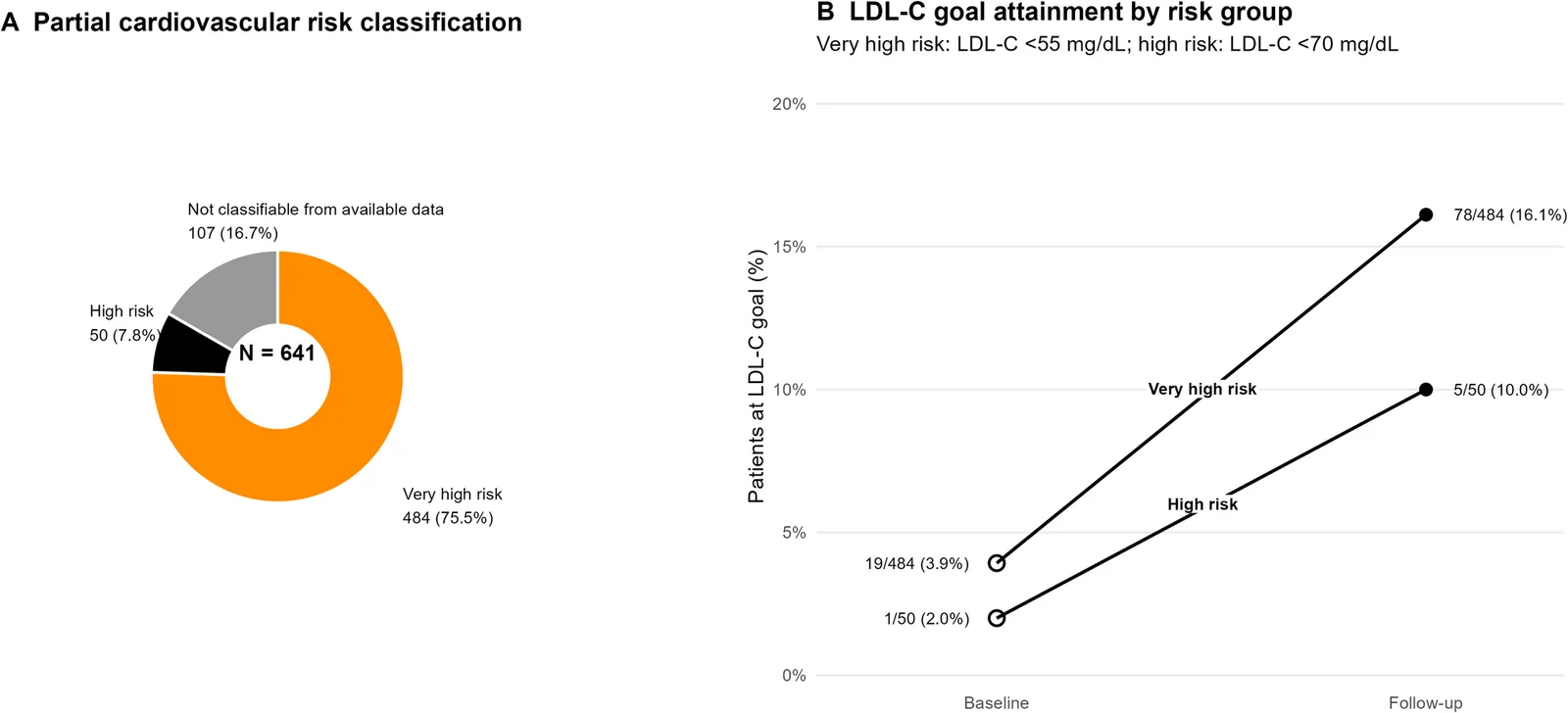
Partial cardiovascular risk classification and LDL-C goal attainment. Panel **A** shows the post hoc partial cardiovascular-risk classification based on unequivocal variables available in the dataset. Panel **B** shows absolute LDL-C goal attainment at baseline and follow-up among classifiable patients (LDL-C <55 mg/dL for very-high-risk and <70 mg/dL for high-risk patients)

Among 484 patients meeting a very-high-risk criterion, 19 (3.9%) had LDL-C < 55 mg/dL at baseline and 78 (16.1%) at follow-up. Among 50 patients meeting a high-risk criterion, 1 (2.0%) had LDL-C < 70 mg/dL at baseline and 5 (10.0%) at follow-up (Fig. 3).

**Fig. 4:**
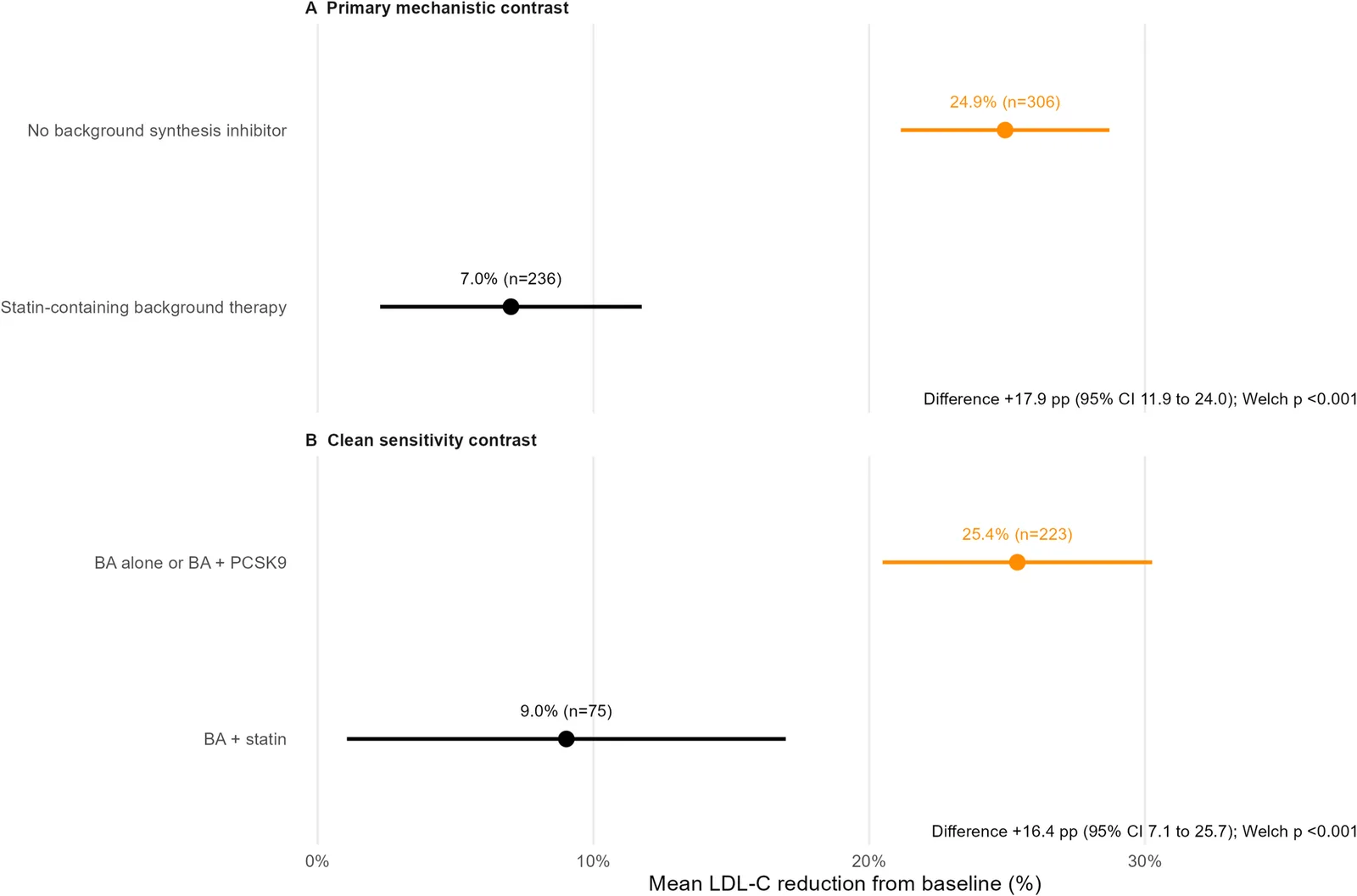
Exploratory LDL-C reduction by background cholesterol-synthesis inhibition. Panel A compares bempedoic acid without a concomitant cholesterol-synthesis inhibitor (no concomitant LLT, ezetimibe or PCSK9 inhibitor) with statin-containing background therapy (statin or statin plus ezetimibe). Panel B shows the restrictive sensitivity comparison of bempedoic acid alone or with PCSK9 inhibition versus bempedoic acid plus statin. Points show mean patient-level LDL-C reduction with 95% confidence intervals

In a post hoc exploratory analysis, mean patient-level LDL-C reduction was 24.9% among patients not receiving a background cholesterol-synthesis inhibitor (no concomitant LLT, ezetimibe or PCSK9 inhibitor; *n* = 306) and 7.0% with statin-containing background therapy (statin or statin plus ezetimibe; *n* = 236), a difference of 17.9% points (95% CI 11.9–24.0; Welch *p* < 0.001; Fig. 4). This association remained significant in endpoint ANCOVA adjusted for baseline LDL-C, age and sex (adjusted follow-up LDL-C 99.4 vs. 109.6 mg/dL; adjusted difference − 10.2 mg/dL, 95% CI -17.1 to -3.4; *p* = 0.004). In the more restrictive sensitivity comparison of bempedoic acid alone or with PCSK9 inhibition versus bempedoic acid plus statin, mean reductions were 25.4% versus 9.0% (difference 16.4% points, 95% CI 7.1–25.7; *p* < 0.001); the adjusted difference was directionally similar but not statistically significant (*p* = 0.064).

**Fig. 5:**
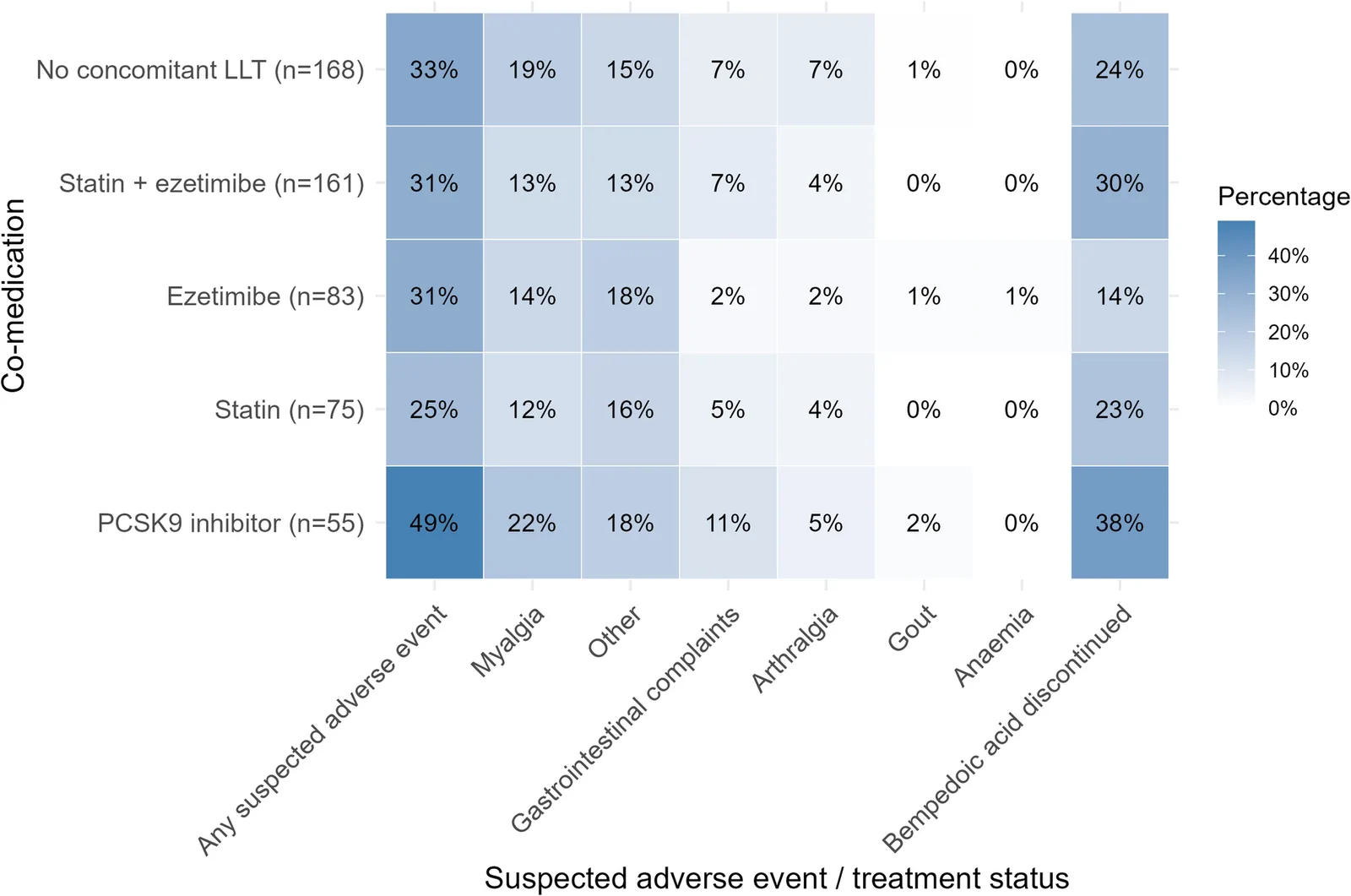
Suspected adverse events at follow-up by co-medication at baseline.*. *Excludes combinations received by fewer than 50 patients. PCSK9i, proprotein convertase subtilisin/kexin type 9 inhibitor

Overall, 34.0% (n = 218) of patients had at least one suspected adverse event documented at follow-up, with myalgia being the most common (16.5% [n = 106]), followed by gastrointestinal complaints (6.7% [n = 43]; Table 4). Arthralgia was recorded in 34 patients (5.3%), gout in 3 (0.5%) and anaemia in 1 (0.2%). An ‘Other’ adverse event was recorded in 109 patients (17.0%); categories were not mutually exclusive. The most frequently repeated exact free-text entries within ‘Other’ were hyperuricaemia (*n* = 3), dizziness (*n* = 3), uric-acid increase (*n* = 2) and headache (*n* = 2), with additional heterogeneous single-patient entries. When analysed by co-medication at baseline, myalgia was the most common adverse event, ranging from 12.0% in patients receiving statins to 22.0% in patients receiving PCSK9is (Fig. 5).

**Table 4:** Suspected adverse events at 3-month follow-up

n (%)	N = 641
Other	109 (17.0)
Myalgia	106 (16.5)
Gastrointestinal complaints	43 (6.7)
Arthralgia	34 (5.3)
Gout	3 (0.5)
Anaemia	1 (0.2)

Patients could have more than one suspected adverse event; categories are not mutually exclusive and percentages should not be summed. 'Other' comprises heterogeneous free-text adverse events not represented by the prespecified categories

In total, 448 patients (69.9%) continued treatment with bempedoic acid at the standard regimen, with a dose reduction and longer dosing interval (e.g. every second day) in 11 (1.7%) and 9 (1.4%) additional patients, respectively. 171 patients (26.7%) discontinued treatment. Myalgia was the most commonly reported adverse event in patients discontinuing treatment (75/171 patients, 43.9%). Treatment status was missing/unknown for 2 patients.

## Discussion

In this real-world study of patients with hypercholesterolaemia or mixed dyslipidaemia, treatment with bempe doic acid alongside other LLTs or without concomitant LLT was associated with a reduction of LDL-C levels of 17.5% on average at the patient level after 3 months of treatment. Furthermore, the greatest unadjusted percentage reductions in LDL-C levels were observed in patients receiving bempedoic acid without concomitant LLT. Similar relative reductions were observed with ezetimibe or PCSK9 inhibitor background therapy, whereas smaller incremental reductions were observed in statin-containing regimens.

This reduction in LDL-C levels is consistent with that observed in other real-world studies [19–21]. In a United Kingdom (UK) multicentre audit of 221 patients with primary hypercholesterolaemia or mixed dyslipidaemia, the mean reduction of LDL-C levels from baseline to 12 weeks after bempedoic acid initiation was 22.0% [19]. In a single-centre, prospective study of 120 patients at high CV risk with LDL-C levels > 70 mg/dL, the addition of bempedoic acid to a statin plus ezetimibe reduced LDL-C levels by 22.9% at 12 weeks [20]. In a study based on German electronic medical records, in patients with no LLT (*n* = 54) and unchanged concomitant LLT (*n* = 371), median LDL-C reductions of 29.9% and 20.3%, respectively, were observed after bempedoic acid initiation [22]. A simulation study conducted in German outpatients at high or very high CV risk found that the sequential addition of ezetimibe and bempedoic acid resulted in a larger proportion of patients achieving guideline-recommended LDL-C goals [23], with similar findings found in the multinational SANTORINI cohort [21].

The pattern according to background therapy is also consistent with randomized evidence. In pooled phase 3 trials, bempedoic acid produced greater placebo-corrected LDL-C lowering in statin-intolerant than in maximally tolerated statin-treated populations [11], and recent reviews and expert guidance discuss its role both without statins and as part of combination therapy [17, 18]. Because bempedoic acid inhibits ATP-citrate lyase upstream of HMG-CoA reductase [10], the smaller incremental response observed with statin-containing background therapy may be compatible with less additional LDL-C lowering when hepatic cholesterol synthesis is already pharmacologically inhibited. Our post hoc mechanism-informed analysis remained significant after adjustment for baseline LDL-C, age and sex in the broader contrast, although the restrictive sensitivity contrast was not statistically significant after adjustment. These findings are therefore hypothesis-generating and do not establish a causal pharmacodynamic interaction.

Absolute LDL-C goal attainment improved but remained low in this clinically challenging cohort: LDL-C < 55 mg/dL increased from 3.9% to 16.1% among patients meeting a very-high-risk criterion, and LDL-C < 70 mg/dL increased from 2.0% to 10.0% among those meeting a high-risk criterion. These figures should be interpreted cautiously because complete cardiovascular-risk classification and untreated LDL-C values were not available for all patients.

Bempedoic acid was associated with suspected adverse events in 34.0% of patients at follow-up, with myalgia being both the most common adverse event and reason for discontinuation, which is in line with observations in a real-world study conducted in Italy [24]. Notably, this cohort is gathered from specialist centres and includes a substantial number of patients experiencing adverse events from statin use, which can lead to medication aversion [25]. No unexpected pattern was apparent in the recorded events, although retrospective ascertainment limits safety inference. Interestingly, several centres attempted to mitigate adverse events by either reducing the dose or extending the bempedoic acid dosing interval. These approaches were used on an individual, compassionate-use basis and are not supported by the approved prescribing information [15]. Further research could clarify whether similar strategies might reduce adverse events and improve adherence in patients receiving bempedoic acid while maintaining clinically relevant lipid-lowering effects.

This retrospective study provides valuable insight into the real-world effectiveness of bempedoic acid in lowering LDL-C levels in routine clinical practice. This study characterises the use of bempedoic acid in a specialist lipid clinic setting, showing its efficacy and limitations in a clinically challenging cohort of patients. Limitations of this approach include the descriptive nature of the study, the short follow-up period, high LLT discontinuation rates at baseline, and unmeasured variables and missing data. Exact treatment-discontinuation dates were not retained, so the temporal relationship between treatment cessation and the follow-up lipid measurement could not be determined for individual patients. Because the analytic dataset did not include a screening log, the number of potentially eligible patients who were not included could not be reconstructed, and selection bias cannot be excluded. However, the use of real-world data enhances the generalisability of these findings to routine clinical practice. Additional data from larger studies with a longer follow-up period will provide further information on the use of bempedoic acid in clinical practice. Additional limitations are that exact treatment-initiation-to-follow-up dates were unavailable; a dedicated HeFH variable, complete formal ESC/EAS risk classification, and reliable statin-intensity classification could not be reconstructed; the phenotype of prior LLT intolerance was not systematically captured; and suspected adverse events were ascertained retrospectively from routine records. Inflammatory biomarkers and other mechanistic endpoints were not systematically collected. The subgroup analyses were performed post hoc and remain susceptible to treatment-selection bias and residual confounding.

## Conclusions

Findings from this real-world, multicentre study conducted in Germany show that bempedoic acid may potentially be an effective treatment option for patients with hypercholesterolaemia who are already receiving LLT, and for patients not receiving or tolerating other LLTs. Responses varied across background treatment strategies, with substantial reductions observed in the absence of concomitant statin therapy. These findings support the effectiveness of bempedoic acid in a real-world, specialist care setting.

## Data Availability

Data are available on reasonable request.

## Abbreviations

ACS: Acute coronary syndrome
ASCVD: Atherosclerotic cardiovascular disease
ANOVA: Analysis of variance
EAS: European Atherosclerosis Society
ESC: European Society of Cardiology
CV: Cardiovascular
HDL-C: High-density lipoprotein cholesterol
ILEP: International Lipid Expert Panel
LDL-C: Low-density lipoprotein cholesterol
LLT: Lipid-lowering therapy
MANOVA: Multivariate analysis of variance
PCSK9i: Proprotein convertase subtilisin/kexin type 9 inhibitor
SD: Standard deviation
TC: Total cholesterol
TG: Triglycerides
UK: United Kingdom
BA: Bempedoic acid

## References

[1] Robert Koch Institut. Cardiovascular diseases. 2025. Available from: https://www.gbe.rki.de/EN/Topics/HealthStatus/PhysicalHealth/CardiovascularDiseases/CardiovascularDiseases_node.html.

[2] Mach F, Baigent C, Catapano AL, Koskinas KC, Casula M, Badimon L, et al. 2019 ESC/EAS Guidelines for the management of dyslipidaemias: lipid modification to reduce cardiovascular risk. Eur Heart J. 2020;41(1):111–88. 31504418 10.1093/eurheartj/ehz455

[3] Ference BA, Braunwald E, Catapano AL. The LDL cumulative exposure hypothesis: evidence and practical applications. Nat Rev Cardiol. 2024;21(10):701–16. 38969749 10.1038/s41569-024-01039-5

[4] Cholesterol Treatment Trialists (CTT), Collaboration, Baigent C, Blackwell L, Emberson J, Holland LE, Reith C, et al. Efficacy and safety of more intensive lowering of LDL cholesterol: a meta-analysis of data from 170,000 participants in 26 randomised trials. Lancet. 2010;376(9753):1670–81. 21067804 10.1016/S0140-6736(10)61350-5 PMC2988224

[5] Banach M, Penson PE, Vrablik M, Bunc M, Dyrbus K, Fedacko J, et al. Optimal use of lipid-lowering therapy after acute coronary syndromes: A Position Paper endorsed by the International Lipid Expert Panel (ILEP). Pharmacol Res. 2021;166:105499. 33607265 10.1016/j.phrs.2021.105499

[6] Mach F, Koskinas KC, van Roeters JE, Tokgozoglu L, Badimon L, Baigent C, et al. 2025 Focused Update of the 2019 ESC/EAS guidelines for the management of dyslipidaemias. Eur Heart J. 2025;46(42):4359–78. 40878289 10.1093/eurheartj/ehaf190

[7] Gouni-Berthold I, Schaper F, Schatz U, Tabbert-Zitzler A, Fraass U, Sauer S, et al. Low-density lipoprotein cholesterol goal attainment in germany: results from the DA VINCI study. Atheroscler Plus. 2022;50:10–6. 36643801 10.1016/j.athplu.2022.07.024 PMC9833225

[8] Banach M, Reiner Z, Surma S, Bajraktari G, Bielecka-Dabrowa A, Bunc M, et al. 2024 Recommendations on the optimal use of lipid-lowering therapy in established atherosclerotic cardiovascular disease and following acute coronary syndromes: a position paper of the international lipid expert panel (ILEP). Drugs. 2024;84(12):1541–77. 39497020 10.1007/s40265-024-02105-5 PMC11652584

[9] Byrne RA, Rossello X, Coughlan JJ, Barbato E, Berry C, Chieffo A, et al. 2023 ESC Guidelines for the management of acute coronary syndromes. Eur Heart J. 2023;44(38):3720–826. 37622654 10.1093/eurheartj/ehad191

[10] Pinkosky SL, Newton RS, Day EA, Ford RJ, Lhotak S, Austin RC, et al. Liver-specific ATP-citrate lyase inhibition by bempedoic acid decreases LDL-C and attenuates atherosclerosis. Nat Commun. 2016;7:13457. 27892461 10.1038/ncomms13457 PMC5133702

[11] Banach M, Duell PB, Gotto AM Jr., Laufs U, Leiter LA, Mancini GBJ, et al. Association of bempedoic acid administration with atherogenic lipid levels in phase 3 randomized clinical trials of patients with hypercholesterolemia. JAMA Cardiol. 2020;5(10):1124–35. 32609313 10.1001/jamacardio.2020.2314 PMC7330832

[12] Nissen SE, Lincoff AM, Brennan D, Ray KK, Mason D, Kastelein JJP, et al. Bempedoic acid and cardiovascular outcomes in statin-intolerant patients. N Engl J Med. 2023;388(15):1353–64. 36876740 10.1056/NEJMoa2215024

[13] Ray KK, Bays HE, Catapano AL, Lalwani ND, Bloedon LT, Sterling LR, et al. Safety and efficacy of bempedoic acid to reduce LDL cholesterol. N Engl J Med. 2019;380(11):1022–32. 30865796 10.1056/NEJMoa1803917

[14] Ballantyne CM, Laufs U, Ray KK, Leiter LA, Bays HE, Goldberg AC, et al. Bempedoic acid plus ezetimibe fixed-dose combination in patients with hypercholesterolemia and high CVD risk treated with maximally tolerated statin therapy. Eur J Prev Cardiol. 2020;27(6):593–603. 31357887 10.1177/2047487319864671 PMC7153222

[15] European Medicines Agency. Bempedoic acid. Summary of product characteristics. 2024. Available from: https://www.ema.europa.eu/en/documents/product-information/nilemdo-epar-product-information_en.pdf.

[16] European Medicines Agency. Bempedoic acid/ezetimibe. Summary of product characteristics. 2024. Available from: https://www.ema.europa.eu/en/documents/product-information/nustendi-epar-product-information_en.pdf.

[17] Strikic D, Begic Z, Radman I, Zlopasa F, Mateljic J, Zec I et al. Reshaping dyslipidaemia treatment with bempedoic acid-a narrative review. Biomedicines. 2025;13(6). 10.3390/biomedicines13061460 PMC12191183 40564178

[18] Banach M, Penson PE, Farnier M, Fras Z, Latkovskis G, Laufs U, et al. Bempedoic acid in the management of lipid disorders and cardiovascular risk. 2023 position paper of the International lipid expert panel (ILEP). Prog Cardiovasc Dis. 2023;79:2–11. 36889490 10.1016/j.pcad.2023.03.001

[19] Ramachandran S, Maarouf A, Mitchell K, Avades T, Smith P, Boulton L, et al. A UK multicentre audit of the management of patients with primary hypercholesterolaemia or mixed dyslipidaemia with bempedoic acid against published lipid-lowering treatment targets. Drugs Context. 2024;13:2024–2. 39165612 10.7573/dic.2024-2-4 PMC11335357

[20] Marazzi G, Caminiti G, Perrone MA, Campolongo G, Cacciotti L, Giamundo DM, et al. Addition of bempedoic acid to statin-ezetimibe versus statin titration in patients with high cardiovascular risk: a single-centre prospective study. J Cardiovasc Dev Dis. 2024;11(9):286. 39330344 10.3390/jcdd11090286 PMC11432672

[21] Katzmann JL, Ray KK, Lee B, Becker C, Articus K, Catapano AL, et al. Oral triple combination lipid-lowering therapy and LDL cholesterol goal attainment: a simulation using the SANTORINI study cohort. Eur J Prev Cardiol. 2025:zwaf777. ahead of print. 10.1093/eurjpc/zwaf777 41436023

[22] Katzmann JL, Becker C, Schneider TF, Articus K, Keuken G, Laufs U. Real-world effectiveness of bempedoic acid in reducing LDL-C: an electronic medical records-based analysis. JACC Adv. 2026;5(5):102726. 41950883 10.1016/j.jacadv.2026.102726 PMC13090588

[23] Katzmann JL, Becker C, Bilitou A, Laufs U. Simulation study on LDL cholesterol target attainment, treatment costs, and ASCVD events with bempedoic acid in patients at high and very-high cardiovascular risk. PLoS ONE. 2022;17(10):e0276898. 36301892 10.1371/journal.pone.0276898 PMC9612573

[24] Russo V, Ratti G, Parrella A, De Falco A, Crisci M, Franco R, et al. clinical utilization and performance of bempedoic acid in an italian real-world setting: Insight from Campania Region. J Clin Med. 2025;14(6):1839. 10.3390/jcm14061839 PMC11943254 40142647

[25] Xie M, Martin SS, Turchin A. Reasons for non-acceptance of statin therapy by patients at high cardiovascular risk. Sci Rep. 2025;15(1):17014. 40379793 10.1038/s41598-025-01930-2 PMC12084616

